# FMRFamide-Like Immunoreactivity in the Central Nervous System and Alimentary Tract of the Non-Hematophagous Blow Fly, *Phormia regina*, and the Hematophagous Horse Fly, *Tabanus nigrovittatus*

**DOI:** 10.1673/031.008.6501

**Published:** 2008-10-27

**Authors:** Aaron T Haselton, Chih-Ming Yin, John G Stoffolano

**Affiliations:** ^1^ Department of Biology, State University of New York at New Paltz, New Paltz, NY; ^2^ Department of Entomology, University of Massachusetts Amherst, Amherst MA

**Keywords:** Retrocerebral complex, stomatogastric nervous system, endocrine cell

## Abstract

FMRFamide-related peptides (FaRPs) are a diverse and physiologically important class of neuropepeptides in the metazoa. In insects, FaRPs function as brain-gut neuropeptides and have been immunolocalized throughout the nervous system and alimentary tract where they have been shown to affect feeding behavior. The occurrence of FMRFamide-like immunoreactivity (FLI) was examined in the central nervous system and alimentary tract of non-hematophagous blow fly, *Phormia regina* Meigen (Diptera: Calliphoridae), and the hematophagous horse fly, *Tabanus nigrovittatus* Macquart (Diptera:Tabanidae). Although the central nervous system and alimentary anatomy differ between these two dipteran species, many aspects of FLI remain similar. FLI was observed throughout the central and stomatogastric nervous systems, foregut, and midgut in both flies. In the central nervous system, cells and processes with FLI occurred in the brain, subesophageal ganglion, and ventral nerve cord. FLI was associated with neurohemal areas of the brain and ventral nerve cord. A neurohemal plexus of fibers with FLI was present on the dorsal region of the thoracic central nervous system in both species. In the gut, processes with FLI innervated the crop duct, crop and anterior midgut. Endocrine cells with FLI were present in the posterior midgut. The distribution of FLI in these two flies, in spite of their different feeding habits, further supports the role of FaRPs as important components of the braingut neurochemical axis in these insects and implicates FaRPs as regulators of insect feeding physiology among divergent insect taxa.

## Introduction

FMRFamide-related peptides (FaRPs) are a diverse family of vertebrate and invertebrate neuropeptides possessing a C-terminal RF-amide amino acid sequence. FaRPs are typified by the first tetrapeptide identified with this defining terminal sequence, the molluscan cardioexcitatory tetrapeptide FMRFamide (Price and Greenberg 1977), and compose an extensive neuropeptide super-family with multiple physiological and behavioral functions in insects. FaRPs can be divided into five major subfamilies based on their C-terminal sequence just upstream of the terminal RF-amide: the extended FMRFamides (to date, the extended FMRFamides have only been identified in the Diptera), the FLRFamides, the HMRFamides, the RLRFamides, and the RVRFamides (Nässel et al. 2002). Immunohistochemical studies have revealed neuronal FaRP-like immunoreactivity (FLI) in most animal groups investigated (Sivasubramanian 1991; Duve et al. 1994). Extensive FLI has been discovered in many insects across distantly related taxa, and the association of FLI with virtually all neuronal types and many peripheral tissues attests to the pivotal role of FaRPs in a multitude of physiological processes and behaviors (Orchard et al. 2001).

FLI has been described in various tissues in many dipteran species, including *Drosophila melanogaster* (White et al. 1986; Nichols 1992b; Nichols 2003), *Calliphora vomitoria* (Lundquist and Nässel 1990; Duve et al. 1994), *C. erythrocephala* (Cantera and Nässel 1991), *Neobellieria* (*Sarcophaga*) *bullata* (Sivasubramanian 1991; Sivasubramanian 1992; Fonagy et al. 1992a; Fonagy et al. 1992b), *Phormia regina* (Richer et al. 2000), *Aedes aegypti* (Brown et al. 1986; Veenstra et al. 1995; Moffet and Moffet 2005), *Hematoma irritans, Stomoxys calcitrans* (Meola et al. 1996), and *Musca domestica* (Agricola and Braunig 1995; Haselton et al. 2004). FLI in the Diptera occurs in cells and processes of the CNS, the stomatogastric nervous system, and in endocrine cells of the midgut. Extensive FLI in the alimentary tract suggests that these peptides play an important role in the regulation of feeding physiology in the Diptera. Evidence for FaRPs as major brain-gut hormones exists in other insect orders Jenkins et al. 1989; Elia et al. 1993; Lange 2001; Hill and Orchard 2004) and several FaRPs have been shown to modulate crop muscle contractions in two species of flies (Nichols 1992a; Richer et al. 2000; Duttlinger et al. 2002). The significance of FaRPs as integral physiological messengers in the Diptera is further indicated by the discovery of multiple, tandem copies of FMRFamide encoding genes in *D. melanogaster* and *C. vomitoria* (Nambu et al. 1988; Duve et al. 1994; Schneider and Taghert 1998).

Hematophagous flies typically have a distensible region of their posterior midgut that houses the ingested blood meal while diuresis and the digestion and absorption of nutrients occurs. This specialized distensible midgut region has not been described in non-hematophagous Diptera. As FLI, and particularly midgut cell FLI, have been described in both hematophagous and non-hematophagous insects, it is possible that the distribution of these peptides may vary somewhat within insects that exhibit these different feeding habits and digestive tract morphologies. Serotonergic innervation has been shown to differ between closely related hematophagous and non-hematophagous insects (Miggiani et al. 1999), and midgut innervation with serotonergic processes differs dramatically between hematophagous and non hematophagous Diptera (Nässel 1988; Moffet and Moffet 2005; Haselton et al. 2006). A comparison of the distribution of cells and processes with FLI within hematophagous and non-hematophagous fly species may provide information regarding the functional diversity of this family of peptides within the Diptera.

In this report, the patterns of FLI are examined in two flies that are well established models for feeding physiology, the queen blow fly, *Phormia regina* Meigen (Diptera:Calliphoridae), and the horse fly, *Tabanus nigrovittatus* Macquart (Diptera:Tabanidae). This is the first study to directly compare both alimentary and CNS FLI patterns between a hematophagous and a non-hematophagous dipteran.

## Materials and Methods

### Animals

*P. regina* were reared and maintained as previously described (Stoffolano 1974). Flies were exposed to a 16:8 L:D photoperiod at 28 ± 2° C, 50 % relative humidity and were provided with granulated sugar and water in their cages, *ad libitum*. Only three-day-old, adult, female flies were used for immunohistochemical investigations.

Host-seeking, adult, female *T. nigrovittatus* were collected from salt marsh box traps on Pine Island in Essex Co., MA. Flies were transferred from the traps to screen cages containing granulated sugar and water and were transported to the laboratory at the University of Massachusetts. In the laboratory, flies were maintained at ambient temperatures and photoperiod with damp towels placed over their cages to provide increased humidity. Flies were maintained in the laboratory for 2–3 weeks.

### Dissection

Flies were injected with 4 % paraformaldehyde in PBS and fixed for 10–15 min prior to dissection to ensure tissue integrity and to allow for easier dissection. The entire CNS and the alimentary tract, from the esophagus to the pyloric sphincter, were dissected in a droplet of fixative and cleaned of attached skeletal muscles and trachea. Extracted and cleaned tissues were further fixed in 4% paraformaldehyde in PBS at 4° C for 24 h prior to immunohistochemical staining.

### Antisera

Anti-FMRFamide antiserum was from Diasorin (www.diasorin.com). Tetramethylrhodamine isothiocyanate conjugated secondary antibody was from Jackson Immunoresearch Laboratories (www.jacksonimmuno.com). Antiserum for preabsorbtion controls were incubated with 100 ***µ*** g/ml FMRFamide (Bachem, www.bachem.com) overnight at 4° C.

### Histochemistry

For whole mount fluorescence immunohistochemistry, the immunohistochemical protocol described by Davis (1987) was followed with several minor modifications. Tissues fixed in 4% paraformaldehyde in PBS were washed in PBST (PBS with 0.5% Triton X-100) six changes, 30 min each, and left in the last wash overnight at 4° C. Washed tissues were then blocked with 10% nonimmune goat serum in PBST (10% normal goat serum/PBST) for 1 h while agitated on a shaker table prior to application of primary antiserum. Tissues were probed with primary antiserum diluted in 10% normal goat serum/PBST (anti-FMRFamide, 1:500) for 72 h at 4° C. Probed tissues were washed in PBST (five changes; 30 min each) and again blocked in 10% normal goat serum/PBST for 1 h with agitation. Tissues were soaked in rhodamine-conjugated secondary antiserum (1:200) for 1 h in darkness and with agitation. Subsequent clearing and mounting steps were conducted in near-darkness so as not to diminish the fluorescence of the secondary antibody label. Thirty-minute washes of 40, 60, and 80% glycerine were used to clear tissues. Cleared and stained tissues were mounted on slides in Vectashield mounting medium (Vector Labs, www.vectorlabs.com) covered with a cover slip, sealed with clear nail polish, and stored at -20°C. Slides were examined using a MRC600 laser confocal microscope (Bio Rad, www.bio-rad.com). Micrographs were processed using Confocal Assistant 3.10 (written by Todd Clark Brelje) and Photoshop 5.0 (Adobe, www.adobe.com). Tissues from 10 flies of both fly species were analyzed in this study.

For paraffin sections, entire *P. regina* heads were removed and immersed in Bouin's fixative for 2–3 h. The heads were then dehydrated, cleared, and embedded in Paraplast® as per standard methods (Humason 1967). Sections were cut at 7 ***µ***m using an American Optical Co. model 820 rotary microtome, stained as described below, dehydrated, mounted on albumenized slides, and dried overnight at room temperature. Tissue sections were mounted in Permount® and covered with a cover slip. Adjacent sections were mounted on alternate slides to facilitate ‘mirror’ staining with two different histological techniques.

Mirror staining of neurosecretory cells in adjacent paraffin sections of *P. regina* heads was performed using peroxidase-antiperoxidase immunohistochemical methods and paraldehyde fuchsin. For paraffin immunohistochemistry, a modified version of Sternberger's peroxidase-antiperoxidase (Sternberger 1979) method was followed. Briefly, tissues were blocked with 1:20 normal goat serum:PBS for 30 min, washed in PBS (two changes; 10 min each), and incubated in 1:1000 anti-FMRFamide antiserum for 24 h at 4°C. Sections were then washed and blocked again as above for 30 min at room temperature and incubated with 1:50 goat antirabbit antiserum for 1 h at 37°C, washed in PBS, and incubated in a 1:400 rabbit **PAP** complex for 30 min at 37°C. Slides were washed in PBS and immersed in freshly prepared 0.05% diaminobenzidine/0.015% H_2_O_2_ in 0.05M Tris-HCL until well developed (30–60 min).

Visualization of neurosecretory cells in embedded and sectioned brains was carried out using paraldehyde fuchsin staining as described by Ewen (1962). Adjacent sections of the same brain were mounted on separate slides, one slide was then stained immunohistochemically and the other with paraldehyde fuchsin. All sections were examined using a Nikon (www.nikon.com) E-600 epifluorescent compound microscope equipped with a SPOT-RT camera system (Diagnostic Instruments, www.diaginc.com). Images were processed as described above. Tissues from six flies were analyzed in this study.

### Results

#### Anatomy

The nervous systems and alimentary tracts of *P. regina* and *T. nigrovittatus* vary somewhat in overall structure. The CNS of *P. regina* is composed of the brain (supra and subesophageal ganglia) and ventral nerve cord with its associated thoracico-abdominal ganglion. This ganglion itself is composed of all ancestral thoracic and abdominal neuromeres fused into one ganglion, a derived characteristic of the higher Diptera (Chapman 1998). The CNS structure of *T. nigrovittatus* differs from that of *P. regina* with incomplete fusion of abdominal and thoracic neuromeres. Instead, a consolidated thoracic ganglion resides in the thorax, composed of thoracic neuromeres and the first abdominal neuromere (Yeates et al. 2002). An abdominal chain of ganglia, composed of the remaining abdominal neuromeres as ganglia, is connected to the thoracic ganglion by the ventral nerve cord and continues into the abdomen.

The divergence in alimentary structure between these two flies occurs primarily in the midgut. In *T. nigrovittatus*, the foregut structure is similar to that in *P. regina*, with a long crop duct branching off of the posterior foregut and leading to a bilobed crop sac in the abdomen. The anterior midgut of *T. nigrovittatus* does not possess the specialized, invaginated proventriculus found in *P. regina*. Instead, two large, lateral caeca branch out anteriorly from the anterior midgut at the midgut/foregut junction. Unlike the *P. regina* midgut, the midgut of *T. nigrovittatus* is divided into two visually distinct regions, the tubular anterior or thoracic midgut and the posterior or abdominal distensible midgut, similar to the midgut of *A. aegypti*, which is also a blood feeder (Brown et al. 1986; Veenstra et al. 1995). The midgut of *T. nigrovittatus* is also linear, with no coils or kinks as seen in *P. regina*.

#### Immunohistochemistry

**Figure 1.**
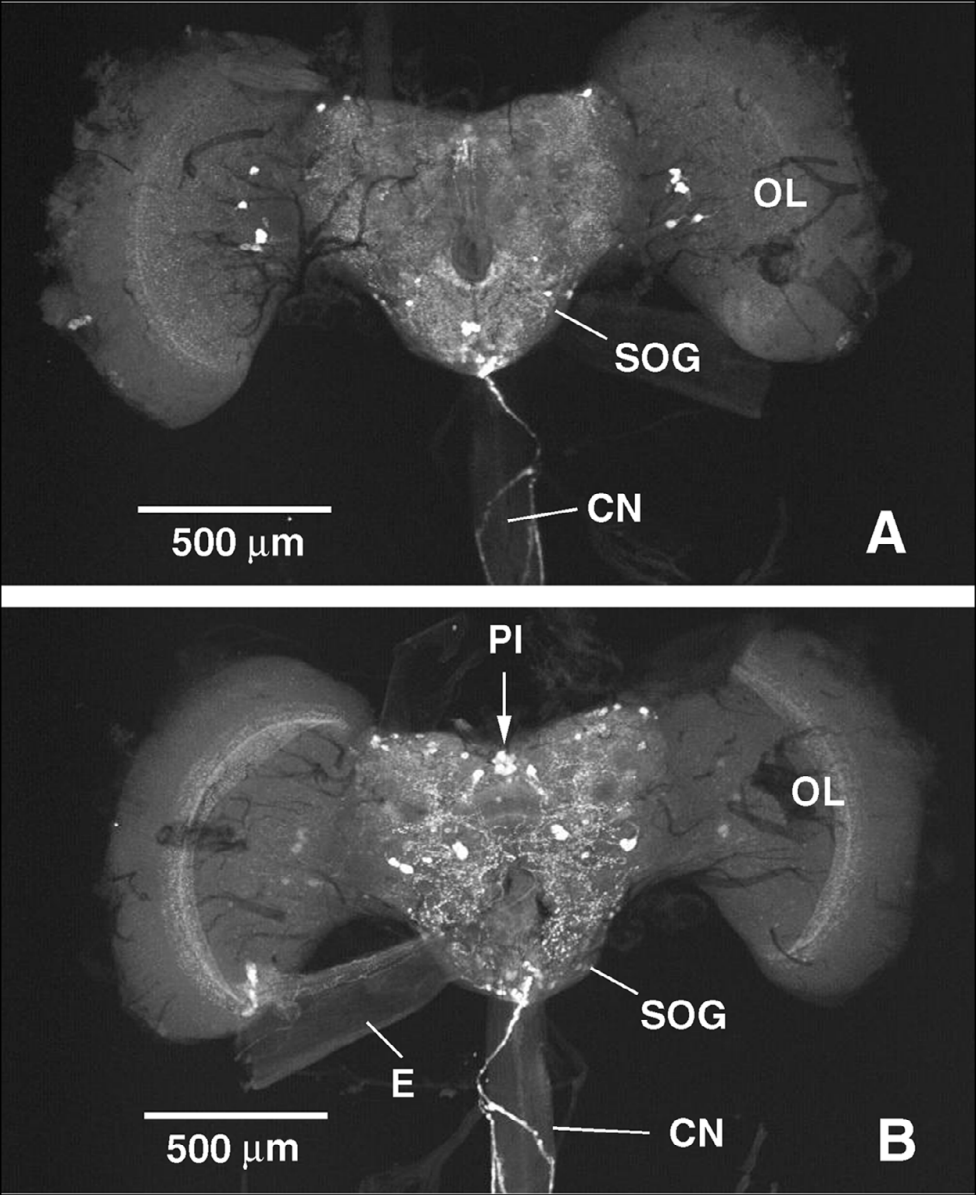
FMRFamide-like immunoreactivity (FLI) in the brain of *Phormia regina*. A) Anterior view of the brain showing FLI throughout the regions of the brain, including cells in the optic lobes, protocerebrum, and the subesophageal ganglion (SOG). FLI is shown in nerve processes running down the cervical connective (CN) to the thoracico-abdominal ganglion. B) Posterior view of the brain showing FLI in cells of the protocerebrum, including median neurosecretory cells in the pars intercerebralis (PI) and subesophageal ganglion, cervical connective processes originate from cells in the subesophageal ganglion. OL, optic lobe; E, esophagus.

Cells exhibiting FLI in the CNS's of both species typically occurred in bilaterally symmetrical pairs or clusters, although the exact number of cells within each cluster was sometimes difficult to determine. Fine, punctate FLI was seen throughout the brain and ganglia of both fly species, particularly in neuropil areas and in integrative centers. Only the most prominent cells and processes with FLI in both flies are described in this report. FLI in the brain of *P. regina* is shown in Figure 1A, B. In the anterior brain of *P. regina*, clusters of cells with FLI with varying staining intensity were observed in the middle and lower proximal regions of the optic lobes. Single, intensely stained cells occurred in the lateral regions of the superior protocerebrum with processes projecting towards the central complex. A cluster of 4 intensely stained cells was observed in the middle region of the subesophageal ganglion. Two intensely stained cells occurred lateral to, and just below, the esophageal foramen, and a cluster of cells were visualized in the ventral midline region of the subesophageal ganglion.

**Figure 2.**
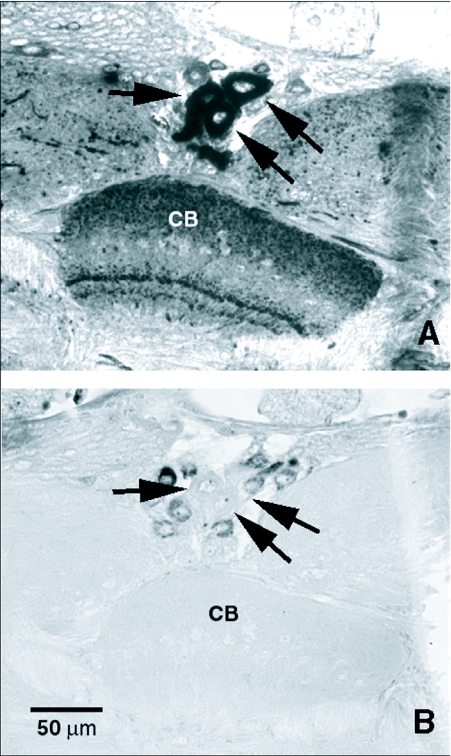
Mirror staining of the pars intercerebralis (PI) of *Phormia regina*. A) pars intercerebralis region of the protocerebrum stained with anti-FMRFamide antiserum using the peroxidase-antiperoxidase technique of Sternberger (1979). Arrows indicate large, medial FMRFamide-like immunoreactive (FLI) cells surrounded by smaller cells exhibiting little or no FLI. B). Adjacent paraffin section showing type-A median neurosecretory cells stained with paraldehyde fuchsin. Arrows indicate large, unstained medial cells corresponding to the FLI cells of the previous section. CB, central body.

The entire posterior surface of the brain was covered by fine, varicose processes with FLI. The posterior aspect of the brain also revealed a central cluster of large cells with FLI in the dorsomedial region of the protocerebrum, just above central complex. Several smaller cells also exhibited FLI on either side of this central cluster in the dorsal region of each protocerebral lobe. The large cells with FLI were nested in the middle of a cluster of smaller median neurosecretory cells, as determined by paraldehyde fuchsin staining (Figures 2A, B). Some of the median neurosecretory cells peripheral to the large cells with FLI also displayed weak FLI. This central cluster of median neurosecretory cells was closely flanked on either side by what appeared to be single large cells with FLI with ventrally directed processes. Pairs of cells lateral to and just above the esophageal foramen, as well as pairs of cells lateral to these, exhibited FLI in the posterior subesophageal ganglion. A cluster of small cells with FLI in the ventral region of the posterior subesophageal ganglion cells was observed, and some of the nerves with FLI running down the cervical connective appeared to originate from this region, possibly from some of these cells.

**Figure 3.**
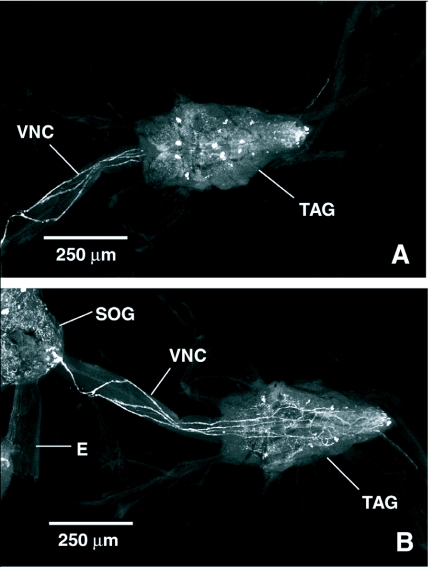
FMRFamide-like immunoreactivity (FLI) in the thoracico-abdominal ganglion (TAG) and cervical connective (CN) of *Phormia regina*. A) Dorsal aspect of the CN and thoracico-abdominal ganglion of *P. regina*. Processes arise from cells in the subesophageal ganglion, run down the cervical connective, and form an anastomosing neurohemal plexus on the dorsum of the thoracico-abdominal ganglion. B) Ventral aspect of the cervical connective and thoracico-abdominal ganglion of *P. regina*. Paired FLI cells and cell clusters are visible in each neuromere of the thoracico-abdominal ganglion. Processes can be seen running down the cervical connective to the thoracico-abdominal ganglion. SOG, subesophageal ganglion; E, esophagus.

Intensely stained nerve tracks run from the subesophageal ganglion, down the cervical connective, and along the dorsal surface of the thoracico-abdominal ganglion where they form a dorsal surface nerve net (Figures 3A, B). Immunoreactive cells were observed in the ventral region of the three thoracic neuromeres and in the abdominal neuromeres (Figure 3B). Each neuromere contained a pair of cells just lateral to the midline with processes projecting towards the dorsal surface of the ganglion. Clusters of small cells were visible just lateral to the larger medial cells. A series of cells with FLI was also visible in the abdominal neuromeres.

**Figure 4.**
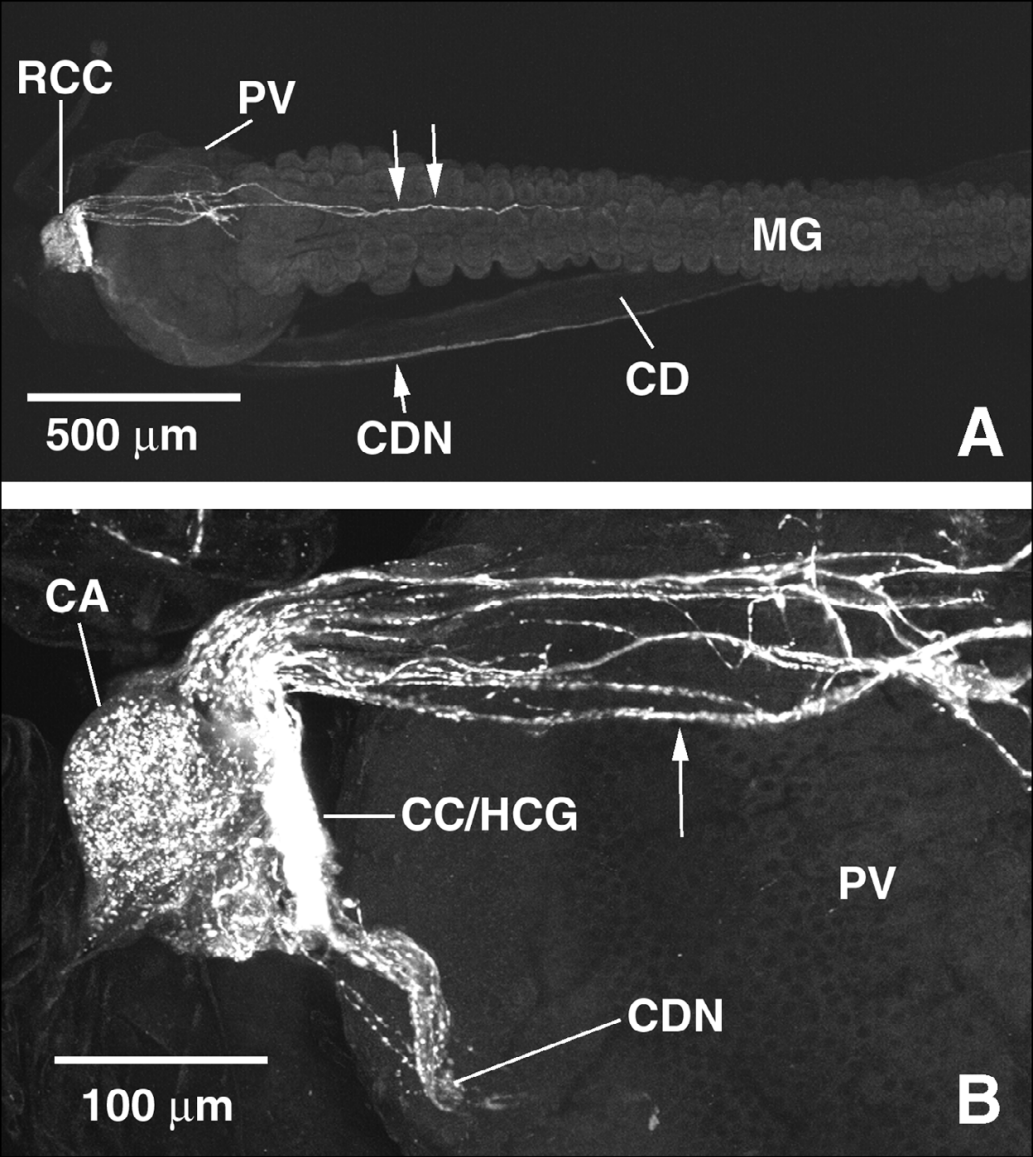
FMRFamide-like immunoreacitivity (FLI) in the retrocerebral complex (RCC) of *Phormia regina*. A) FLI in the retrocerebral complex and anterior dorsal surface of the midgut of *P. regina*. FLI processes originating from the retrocerebral complex run over the proventriculus (PV) and down the dorsal surface of the anterior midgut (double arrows), as well as down the lateral sides of the crop duct (CD) forming two crop duct nerves (CDN). B) An enlarged view of FLI in the retrocerebral complex of *P. regina* shows intense FLI in the corpus cardiacum/hypocerebral ganglion, as well as in the corpus allatum. Processes originating in this intense area of FLI run over the proventriculus to the midgut and down the crop duct to the crop. MG midgut; CA, corpus allatum; CC/HCG, corpus cardiacum/hypocerebral ganglion.

FLI was observed in the retrocerebral complex, including the corpus allatum (CA), and the anterior stomatogastric nervous system of *P. regina*. A dense tangle of processes with FLI underlays the corpus allatum in the region of the corpus cardiacum/hypocerebral ganglion (CC/HCG) (Figures 4A, B). From this mass of processes, two nerve tracts, each with multiple varicose processes, arise and run down the crop duct on either side (Figures 4A, B). Several other unconsolidated fibers arose from the CC/HCG complex, continued over the dorsal side of the proventriculus, and ran along the dorsal surface of the anterior midgut (Figures 4A, B). The CA itself was intensely stained with punctate FLI (Figure 4B).

**Figure 5.**
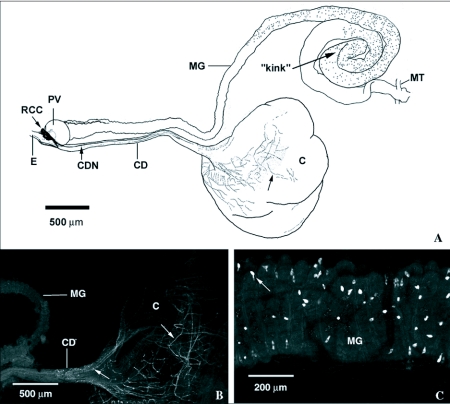
FMRFamide-like immunoreactivity (FLI) in the foregut and midgut of *Phormia regina*. A) Diagram showing FLI in the gut of *P. regina*. FLI occurs in the retrocerebral complex (RCC), crop duct nerve (CDN), and on the proximal surface of the crop sac (C) of the foregut. In the midgut, FLI occurs in nerves running down the dorsal surface of the anterior midgut and in numerous midgut endocrine cells of the posterior midgut. B) FLI processes from the crop duct nerves ramify out into a fine network on the proximal surface of the crop sac (arrows). C) FLI midgut endocrine cells of the posterior midgut. Open-type endocrine cell morphology (apical processes projecting toward lumen) is evident in many cells (arrow). E, esophagus; PV, proventriculus; CD, crop duct; MT, Malpighian tubules.

FLI was studied in the posterior foregut and entire midgut of *P. regina* (Figures 5A, B, C). The two nerve trunks that arose from the CC/HCG region and ran down the lateral sides of the crop duct ramified into a fine, varicose nerve network on the surface of the crop sac (Figures 5A, B). Endocrine cells with FLI occurred only in the posterior region of the midgut. The distribution of these cells began approximately after the anterior ⅓ of the midgut, just anterior to the abdominal helicoid region, and they continued through the helicoid region ‘kink’, but they did not reach the pyloric sphincter. These midgut endocrine cells exhibited open-type, receptosecretory morphology, with thin apical projections oriented towards the lumen (Figure 5C) (Endo and Nishiitsutsuji-Uwo 1981).

**Figure 6.**
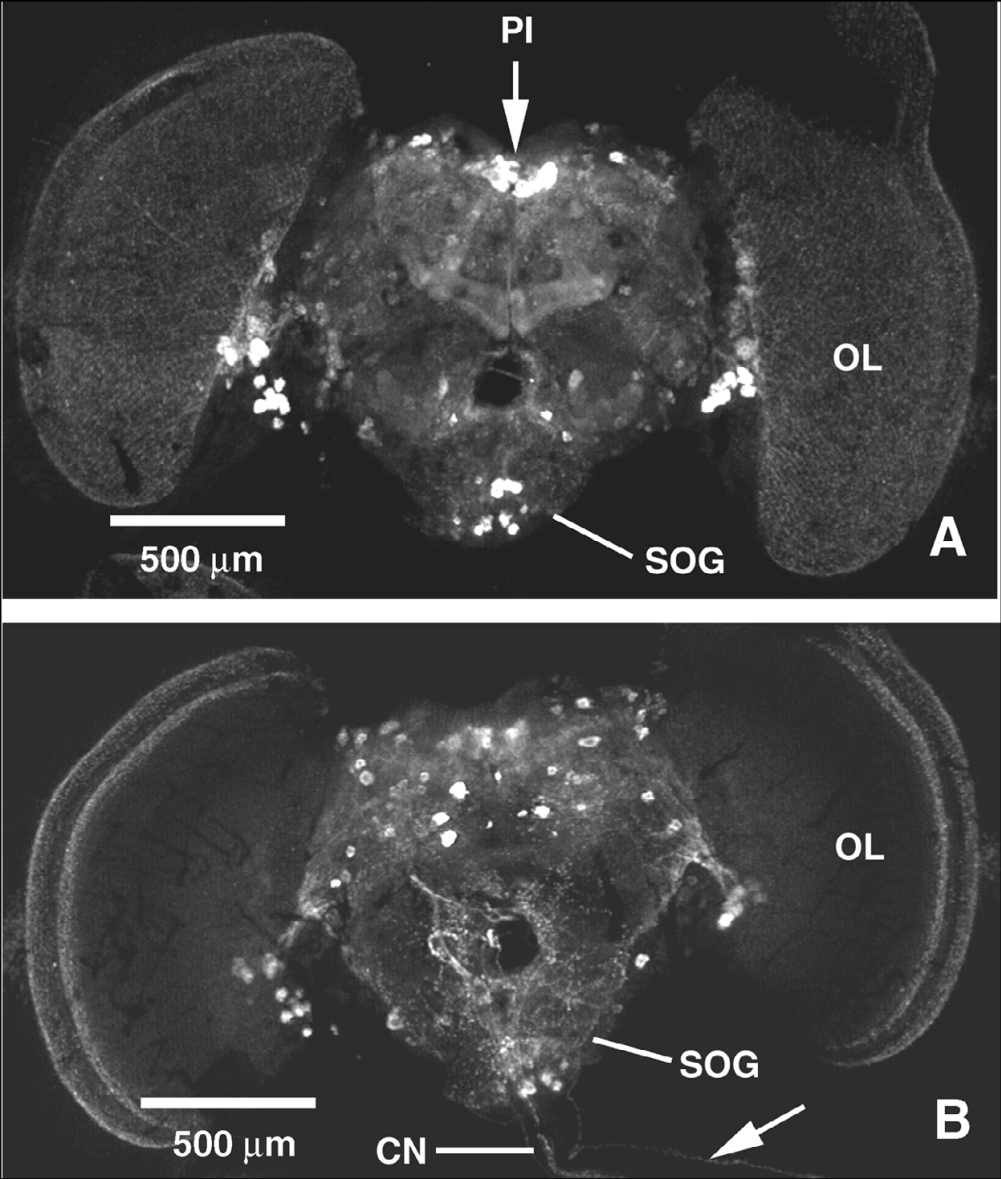
FMRFamide-like immunoreactivity (FLI) in the brain of *Tabanus nigrovittatus*. A) Anterior view of the brain showing FLI throughout the regions of the brain, including cells in the protocerebrum, pars intercerebralis (PI), optic lobes (OL) and the subesophageal ganglion (SOG). B) Posterior view of the brain showing FLI in cells of the protocerebrum and in nerve processes running down the cervical connective (CN). At least some cervical connective processes originate from cells in the subesophageal ganglion.

The brain of *T. nigrovittatus* showed similar FLI to that observed in *P. regina* (Figures 6A, B). Cell bodies with FLI occurred in the median and lateral regions of the superior protocerebrum and more ventrally at the junction of the optic lobes. A cluster of cells with FLI was also visualized in the center of the subesophageal ganglion. The posterior surface of the brain was covered with fine, punctate nerves similar to those observed in *P. regina* and at least some of these processes appeared to be continuous with nerves running down the dorsal surface of the cervical connective.

**Figure 7.**
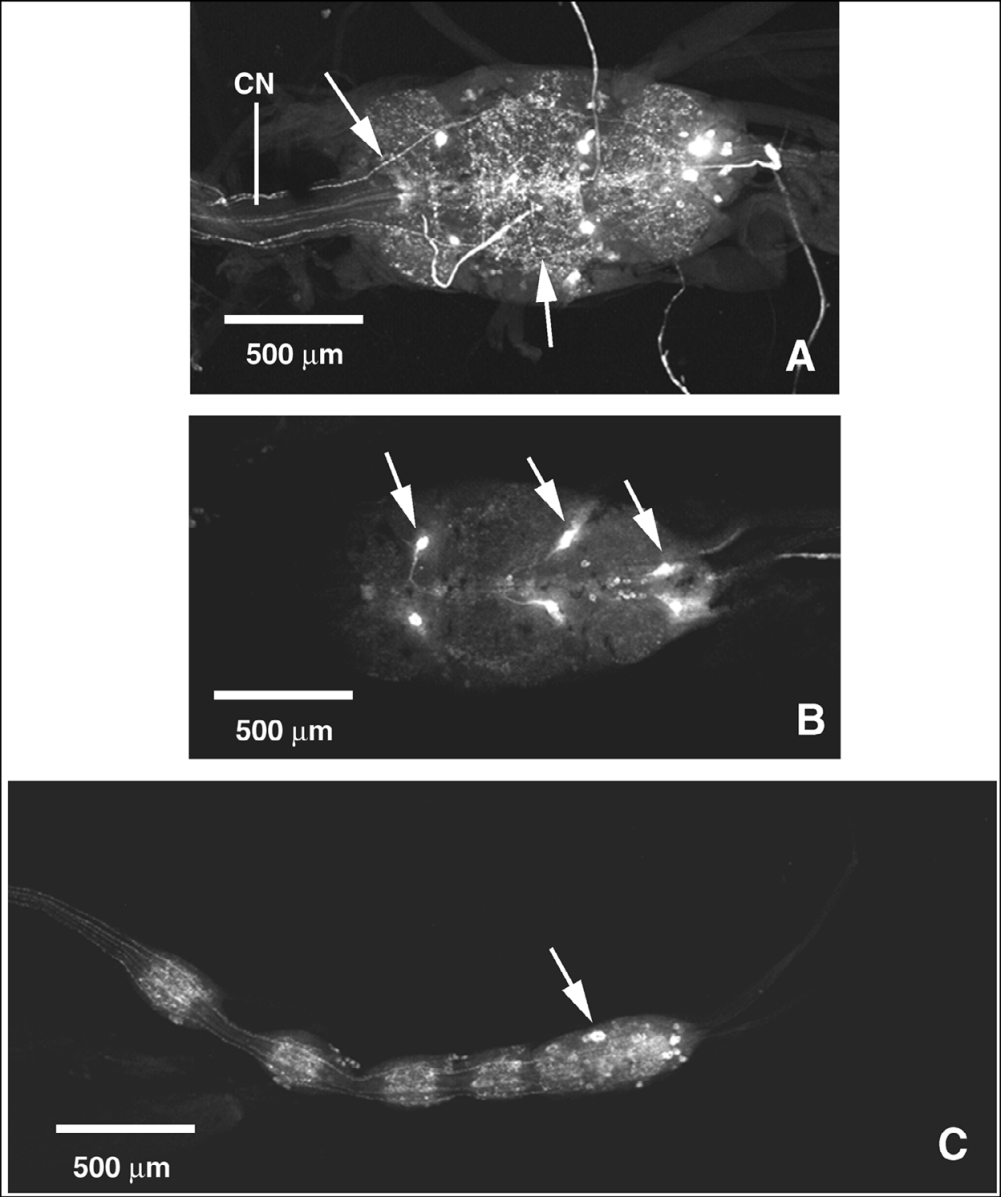
FMRFamide-like immunoreactivity (FLI) in the cervical connective (CN), thoracic ganglion, and abdominal chain ganglia of *Tabanus nigrovittatus*. A) FLI processes running down the cervical connective and over the dorsal surface of the thoracic ganglion (arrows). B) Large, paired FLI cells and cell clusters in the ventral region of each neuromere in the thoracic ganglion (arrow). C) FLI processes run down the entire length of the abdominal chain of ganglia and FLI cell bodies and neuropil occur in each neuromere.

Multiple nerves with FLI ran down the cervical connective and over the dorsal surface of the thoracic ganglion (Figure 7A). The neuromeres of the thoracic ganglion contained multiple, paired cells and cell clusters with FLI in their ventral regions (Figure 7B). Fine, punctate nerves with FLI from the dorsal surface of the thoracic ganglion ran continuously down the length of the chain of ganglia, and each of the five ganglia in the chain possessed multiple cell bodies with FLI, with the most numerous occurring in the terminal neuromere (Figure 7C).

**Figure 8.**
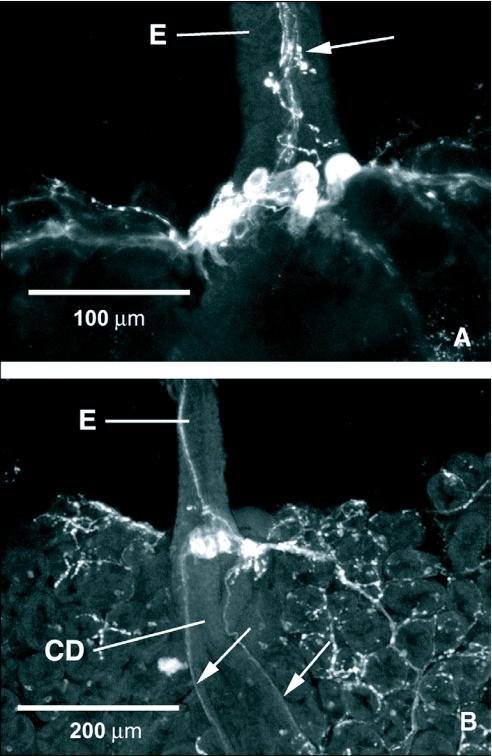
FMRFamide-like immunoreactive (FLI) cells at the foregut/midgut junction in *Tabanus nigrovittatus*. A) A cluster of large FLI cells rest on the dorsal surface of the foregut just anterior to the junction with the midgut. Processes that appear to be associated with these cells form a network that innervates the entire outer surface of the anterior midgut. Punctate FLI processes on the esophagus also communicate with the FLI cell bodies (arrow). B) Two nerves originating from the region of the FLI cell bodies at the foregut/midgut junction run down the lateral sides of the crop duct (arrows). E, esophagus; CD, crop duct.

Several large cell bodies with FLI were observed on the dorsal side of the foregut/midgut junction (Figures 8A, B). Processes with FLI originating in the area of these large cells formed two nerves that ran down the lateral sides of the crop duct (Figure 8B). Punctate processes running down the esophagus appeared to also communicate with these gut cells with FLI (Figures 8A, B).

**Figure 9.**
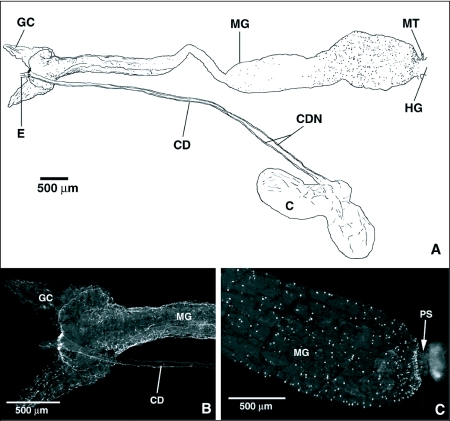
FMRFamide-like immunoreactivity (FLI) in the foregut and midgut of *Tabanus nigrovittatus*. A) diagram showing FLI in the gut of *T. nigrovittatus*. In the foregut, FLI occurs in cells at the junction of the foregut and the midgut, in the crop duct nerves, and on the proximal surface of the crop sac of the foregut. In the midgut, FLI occurs in nerves running along the surface of the anterior midgut and in the midgut endocrine cells of the posterior midgut. FLI in the anterior and posterior midgut of *T. nigrovittatus*. B) The entire surface of the anterior midgut is covered by a fine plexus of FLI fibers. Two large FLI nerves emanating from the region of FLI cells and processes at the foregut/midgut junction run posteriorly on the dorsal surface of the anterior midgut. FLI nerves originating from this same region run down the crop duct (CD). C) FLI midgut endocrine cells in the posterior midgut. FLI midgut endocrine cells exhibit open-type endocrine cell morphology and form a dense ring around the terminal end of the midgut, just anterior to the pyloric sphincter. GC, gastric caecum; MG, midgut; E, esophagus; PS, pyloric sphincter; CDN, crop duct nerve; C, crop; MT, Malpighian tubules; HG, hindgut.

FLI in the foregut and midgut of *T. nigrovittatus* is shown in Figures 9A, B, C. The crop of *T. nigrovittatus* shows similar FLI to that observed in *P. regina*, with two nerves running down the lateral sides of the crop duct and fine punctate processes covering the crop sac surface. The outer surface of the anterior midgut was extensively innervated by a fine network of processes with FLI (Figures 9A, B). Two large nerve trunks with FLI originating from the area of the cells with FLI at the foregut/midgut junction ran posteriorly over the dorsal surface of the anterior midgut and continued down the lateral sides of the tubular midgut. Midgut endocrine cells with FLI were of the open type and were restricted to the distensible posterior midgut (Figures 9A, C). The endocrine cells were distributed evenly throughout the distensible region, except in the area just anterior to the pyloric sphincter, where they were more densely clustered and gave the appearance of a ring of cells (Figure 9C). Preabsorbed controls for both flies revealed no immunoreactivity.

### Discussion

The localization of FLI reported in this paper is subject to all of the limitations of immunohistochemistry, and this is particularly true in this study as the antigen bearing peptide belongs to a large superfamily of structurally similar peptides (Hokfelt et al. 1980). All immunohistochemistry reported here was performed using a commercially available polyclonal antiserum that recognizes the common C-terminal RFamide structure and therefore does not discriminate among individual FaRPs (see White et al. 1986; Nichols 2003). Consequently, FLI reported here in these two fly species may be due to the recognition of individual FaRPs from any of the major FaRP subfamilies, or from the recognition of a combination of colocalized FaRPs from the same or different subfamilies within the same cell. Colocalization of different FaRPs may be widespread in insects and direct evidence for the colocalization of heterogeneous FaRPs within the same cells has previously been obtained from *D. melanogaster*, with three drosulfakinin peptides being processed and expressed in many of the same neurons and drosulfakinins colocalized with FMRFamides (Nichols and Lim 1996; Nichols et al. 1997).

The anti-FMRFamide antiserum used in this study revealed many immunoreactive cells and processes throughout the CNS, the stomatogastric nervous system, and the anterior alimentary tracts of both *P. regina* and *T. nigrovittatus*. This combined nervous/visceral peptide distribution pattern is common in insects, and many neuropeptides are part of the brain-gut axis that serves as a link between the endocrine system of the digestive tract, the neuroendocrine system, and the CNS (Orchard et al. 2001).

The distribution of FLI in the nervous systems of *P. regina* and *T. nigrovittatus* are similar to each other and to those reported in other insects. Both dipterans possessed widespread FLI in cells and processes of all cephalic and bodily ganglia of the CNS. Large cells with FLI were observed were observed in the brains of both species in the pars intercerebralis region and in the lateral regions of the superior protocerebrum, areas known to contain neurosecretory cells in insects (Raabe 1982). FLI has been reported in these same neurosecretory cell regions in the brains of *Carausius morosus* (Miksys et al. 1997), *Rhodnius prolixus* (Tsang and Orchard 1991), and *D. melanogaster* (White et al. 1986). Tu (2000) discovered that type-A median neurosecretory cells (as determined by paraldehyde fuchsin staining) of the pars intercerebralis in *P. regina* are the likely targets of a putative midgut hormone released from midgut endocrine cells following a protein meal (Yin et al. 1994). These median neurosecretory cells exhibit an increase in cell volume and a change in the distribution of stainable materials in the first 12 h after a liver meal (Tu 2000). Elia et al. (1993) suggested that brain median neurosecretory cells in *R. prolixus* might release FaRPs into circulation via brain neurohemal organs (CC/CA) after gorging on a blood meal to stimulate molting and/or ecdysteroid production; the release of FaRPs is presumably in response to a signal from the gut. To determine if the type-A median neurosecretory cells's of the pars intercerebralis in *P. regina* were the same cells exhibiting FLI, mirror staining of adjacent paraffin sections with paraldehyde fuchsin and anti-FMRFamide antiserum was performed. Our findings agree with those found in the *Drosophila* (White et al. 1986) in which only some of the median neurosecretory cells show FLI, indicating that these cells are heterogeneous in their biochemical differentiation. While the largest median cells with FLI visualized in the brain of *P. regina* were not among the type-A median neurosecretory cells, it is still possible that these brain cells with FLI respond to midgut hormone in a manner similar to their neighboring cells and that FaRPs play a role in this neuroendocrine cascade involving midgut hormone, possibly co-releasing with the putative median neurosecretory cells hormone from the CC/CA neurohemal tissues.

Multiple processes with FLI were observed in the cervical connective joining the brain and the thoracic ganglia in both species, and these processes with FLI continued down the ventral abdominal chain of ganglia in *T. nigrovittatus*. Each thoracic neuromere of the thoracico-abdominal ganglion in *P. regina* and the thoracic ganglion in *T. nigrovittatus* contained symmetrical pairs of large cells with FLI and cell clusters similar to those described in other dipterans as the ventral thoracic neurosecretory cells (White et al. 1986; Lundquist and Nässel 1990; Sivasubramanian 1991; Duve et al. 1992; Nässel et al. 1994). Processes with FLI originating in the brain and running down the dorsal region of the cervical connective formed an anastomosing network on the dorsal surfaces of the thoracico-abdominal ganglion in *P. regina* and the thoracic ganglion in *T. nigrovittatus.* The dorsal region or dorsal neural sheath of the thoracic portion of the ventral nerve cord, supplied also by the ventral thoracic neurosecretory cells, has been described as a neurohemal plexus in *C. vomitoria* (Nässel et al. 1994) and may be the site of release for multiple neuromessenger molecules. Messenger molecules released from this neural plexus could enter the hemolymph adjacent to the anterior midgut in close proximity to the dorsal vessel and may therefore have systemic hormonal effects within the insect and/or act as a local paracrine modulator of gut activity.

The association of FaRPs with the stomatogastric nervous system is conserved within the insects (Hill and Orchard 2004) and FLI has been reported in the retrocerebral complex of several dipteran species (White et al. 1986; Sivasubramanian 1992; Veenstra et al. 1995; Meola et al. 1996; Richer et al. 2000; Haselton et al. 2004). The retrocerebral complex of *P. regina* is situated at the junction of the foregut and the midgut and consists of the CA and the CC, the brain neurohemal organs, and the hypocerebral ganglion which is part of the stomatogastric nervous system. Intense FLI was observed in processes surrounding the CC/HCG and in the CA in *P. regina*. Processes with FLI projected from this area of intense immunoreactivity and continued over the proventriculus to the dorsal surface of the anterior midgut and down the crop duct. A similar pattern of FLI has been described in the retrocerebral complex of *R. prolixus* where many cells bodies with FLI were observed in the hypocerebral ganglion and fine interlaced fibers formed a network over the entire retrocerebral complex (Elia et al. 1993; Tang and Ward 1998). Elia et al (1993) also noted that retrocerebral complex FLI decreased dramatically following a blood-meal in *R. prolixus*, and that this decrease corresponded with a pulsatile release of FaRPs into the hemolymph. In *C. morosus*, as well, all evidence gathered to date supports the notion that FaRPs are synthesized in brain neurosecretory cells and transported to the CC for release (Miksys et al. 1997). Similarly, Meola et al. (1996) reported FLI in neurosecretory cell bodies of the HCC and in nerve tracts running from the hypocerebral ganglion to the aorta in *H. irritans* and *S. calcitrans*. FLI was also found in external nerves of the CA and in processes near the dorsal surfaces of the CC in both flies, as well as in nerves running over the dorsal surface of the PV in *S. calcitrans*. Both starved and fed *Heliothis zea*, however, exhibited no statistically significant differences in the locations or quantities of FLI material in the cerebral nervous system, including the CC (Jenkins et al. 1989). Except for a general description of the CC (Woodring and Huffman 1994), the retrocerebral complex in Tabanid flies has not been well characterized. Several FLI cell bodies associated with a dense array of processes with FLI were observed at the foregut/midgut junction in *T. nigrovittatus*. Based on the similarity of the location of these peptidergic structures with retrocerebral complex structures in other dipterans, it is probable that these structures are part of the retrocerebral complex in this fly, possibly the hypocerebral ganglion.

It is likely that the punctuate FLI associated with the CA in *P. regina* is actually in a fine nerve network covering the surface of this neuroendocrine gland similar to the FLI reported in the flies *R. prolixus, H. irritans*, and *S. calcitrans* and in the cockroach *Diploptera punctata* (Elia et al. 1993; Meola et al. 1996, Stay et al. 2003). This FLI innervation may serve to modulate the activities of this gland, including the production and/or release of juvenile hormone as demonstrated in *D. punctata* (Stay et al. 2003). It is possibe that feeding-induced RFamide release triggers the CA to produce or release juvenile hormone, possibly for oogenesis, in the blow fly.

Processes with FLI innervate the diverticulated crops of both flies studied here. In *P. regina*, two crop duct nerves showing FLI originate in the retrocerebral complex, run down the crop duct, and spread out into a fine network over the proximal surface of the crop sac. The innervation of the crop duct and crop sac is similar in *T. nigrovittatus*, with the duct nerve tracts originating in the region of FLI on the dorsal surface of the foregut/midgut junction. FLI in foregut tissues has been described in several fly species including *P. regina* and the *in situ* myotropic effects of several FaRPs have been demonstrated in *P. regina* and *D. melanogaster* crop preparations (Richer et al. 2000; Duttlinger et al. 2002; Haselton et al. 2004). It is therefore likely that FLI in the foregut of *T. nigrovittatus* is indicative of a similar foregut myoregulatory role for FaRPs in this species.

Midgut endocrine cells with FLI were restricted to the posterior region of the midgut in both species and the morphology of the individual cells was typical of that described in other insects. Midgut endocrine cells with FLI having varying spatial distribution throughout the midgut wall have been reported in many insects, including *L. migratoria* (Hill and Orchard 2004), *H. zea* (Jenkins et al. 1989), *R. prolixus* (Tsang and Orchard 1991), *A. aegypti* (Brown et al. 1986; Moffet and Moffet 2005) and *N. bullata* (Sivasubramanian 1992). Insect midgut endocrine cells, like their vertebrate homologues, are divided into two morphological types: open type endocrine cells and closed type endocrine cells. Open-type endocrine cells are teardrop shaped with blunt basal ends and apical cellular extensions projecting towards the lumen, whereas closed type endocrine cells do not possess extensions that contact the lumen (Endo and Nishiitsutsuji-Uwo 1981; see Lange 2001). Insect gut endocrine cells with FLI tend to be of the open variety (Orchard et al. 2001). In both *P. regina* and *T. nigrovittatus*, midgut FLI patterns indicate that FaRPs are synthesized and stored in the posterior midgut where they may exert there effects on nearby cells as paracrine messengers and/or be released into the hemolyph when cells are appropriately stimulated by ingested food as it makes the final portion of its passage through the midgut. While post-feeding changes in midgut FLI were not examined in the present study, Stoffolano et al. (1989) observed the formation of omega bodies in endocrine cells of the posterior midgut and the release of stored granules into the hemolymph from these cells after a liver meal in *P. regina*. It is possible that FaRPs are released from midgut endocrine cells in both *P. regina* and *T. nigrovittatus* after feeding, and that these peptides function as paracrine/endocrines that function to inform neighboring and/or distant tissues about the nutritional state of these insects. Based on the relatively conserved distribution of these FLI midgut cells in these two species, the functions of these neuromessengers in these two insects are likely similar and probably involve fundamental motility and digestive processes.

The extensive FLI observed in the neuropil of every insect investigated to date strongly suggests that FaRPs are important neurotransmitters/neuromodulators throughout the insect central and stomatogastric nervous systems. It is clear from the findings presented in this report that FaRPs are crucial regulatory molecules in the both the non-blood feeder, *P. regina*, and the blood feeder, *T. nigrovittatus*. The widespread FLI in the nervous systems and alimentary tracts suggests that FaRPs are part of the brain-gut axis of neuropeptides in these two fly species. FLI in the neuropil and other processes in the CNS and stomatogastric nervous system ganglia indicate a neurotransmitter/neuromodulator role for FaRPs in these flies and the additional presence of FLI in the CC/CA, dorsal sheath, and midgut endocrine cells suggests that FaRPs are also major circulatory hormones in these insects.

## Abbreviations

CC/CA: corpus cardiacum-corpus allatum complex
CC/HCG: corpus cardiacum-hypocerebral ganglion complex,
CNS: central nervous system,
FaRP: FMRFamide-related peptide,
FLI: FMRFamide-like-immunoreactivity

## References

[bibr01] Agricola HJ, Braunig P, O Breidbach, W Kutsch (1995). Comparative aspects of peptidergic signaling pathways in the nervous systems of arthropods.. *The nervous systems of invertebrates: an evolutionary and comparative approach*.

[bibr02] Brown MR, Crim JW, Lea AO (1986). FMRFamide- and pancreatic polypeptide-like immunoreactivity of endocrine cells in the midgut of a mosquito.. *Tissue and Cell*.

[bibr03] Cantera R, Nässel DR (1991). Dual peptidergic innervation of the blowfly hindgut: a light and electron microscopic study of FMRFamide and proctolin immunoreactive fibers.. *Comparative Biochemistry and Physiology*.

[bibr04] RF Chapman (1998). *The Insects: Structure and Function*..

[bibr05] Davis NT (1987). Neurosecretory neurons and their projections to the serotonin neurohemal system of the cockroach *Periplaneta americana* (L.), and identification of mandibular and maxillary motor neurons associated with this system.. *Journal of Comparative Neurology*.

[bibr06] Duttlinger A, Berry K, Nichols R (2002). The different effects of three *Drosophila melanogaster* dFMRFamide-containing peptides on crop contractions suggest these structurally related peptides do not play redundant functions in gut.. *Peptides*.

[bibr07] Duve H, Johnsen AH, East P, Thorpe A, Davey KG, Peter RE, Tobe SS (1994). Comparative aspects of the FMRFamides of blowflies: isolation of the peptides, genes, and functions.. *XIIth International Congress of Comparative Endocrinology*.

[bibr08] Duve H, Johnsen AH, Sewell JC, Scott AG, Orchard I, Rehfeld JF, Thorpe A (1992). Isolation, structure, and activity of -Phe-Met-Arg-Phe-NH2 neuropeptides (designated calliFMRFamides) from the blowfly *Calliphora vomitoria*.. *Proceedings of the National Academy of Sciences USA
*.

[bibr09] Elia AJ, Tebrugge VA, Orchard I (1993). The pulsatile appearance of FMRFamide-related peptides in the haemolymph and loss of FMRFamide-like immunoreactivity from neuroheamal areas of *Rhodnius prolixus* following a blood meal.. *Journal of Insect Physiology*.

[bibr10] Endo Y, Nishiitsutsuji-Uwo J (1981). Gut endocrine cells in insects: the ultrastructure of the gut endocrine cells of the lepidopterous species.. *Biomedical Research*.

[bibr11] Ewen AB (1962). An improved aldehyde fuchsin staining technique for neurosecretory products in insects.. *Transactions of the American Microscopy Society*.

[bibr12] Fonagy A, Schoofs L, Proost P, Damme JV, Beuds H, Loof AD (1992a). Isolation, primary structure and synthesis of neomyosuppressin, a myoinhibiting neuropeptide from the grey flesh fly, *Neobellieria bullata*.. *Comparative Biochemistry and Physiology
*.

[bibr13] Fonagy A, Schoofs L, Proost P, Damme JV, Loof AD (1992b). Isolation and primary structure of two sulfakinin-like peptides from the flesh fly, *Neobellieria bullata*.. *Comparative Biochemistry and Physiology
*.

[bibr14] Haselton AT, Yin C-M, Stoffolano JG (2006). Occurrence of serotonergic immunoreactivity in the central nervous system and midgut of adult female *Tabanus nigrovittatus* (Diptera: Tabanidae).. *Journal of Medical Entomology*.

[bibr15] Haselton AT, Stoffolano JG, Nichols R, Yin C-M (2004). Peptidergic innervation of the crop and the effects of an ingested nonpeptidal agonist on longevity in female *Musca domestica* (Diptera: Muscidae).. *Journal of Medical Entomology*.

[bibr16] Hill SR, Orchard I (2004). The influence of diet and feeding state on FMRFamide-related peptides in the gut of *Locusta migratoria* L.. *Peptides*.

[bibr17] Hokfelt T, Johansson O, Ljungdahl A, Lundberg JM, Schultzberg M (1980). Peptidergic neurons.. *Nature*.

[bibr18] Humason G (1967). *Animal Tissue Techniques.*.

[bibr19] Jenkins AC, Brown MR, Crim JW (1989). FMRF-amide immunoreactivity and the midgut of the corn earworm (*Heliothis zea*).. *Journal of Experimental Zoology*.

[bibr20] Lange AB (2001). Feeding state influences the content of FMRFamide and tachykinin related peptides in endocrine-like cells of the midgut of *Locusta migratoria*.. *Peptides
*.

[bibr21] Lundquist T, Nässel DR (1990). Substance P, FMRFamide, and gastrin/cholecystokinin-like immunoreactive neurons in the thoracicoabdominal ganglia of the flies *Drosophila* and *Calliphora*.. *Journal of Comparative Neurology
*.

[bibr22] Meola SM, Wright MS, Nichols R, Pendleton MW (1996). Localization of Myosuppressinlike peptides in the hypocerebral ganglion of two blood-feeding flies: horn fly and stable fly (Diptera: Muscidae).. *Journal of Medical Entomology*.

[bibr23] Miggiani L, Orchard I, TeBrugge V (1999). The distribution and function of serotonin in the large milkweed bug, *Oncopeltus fasciatus*: a comparative study with the blood-feeding bug, *
Rhodnius prolixus*.. *Journal of Insect Physiology
*.

[bibr24] Miksys S, Lange AB, Orchard I, Wong V (1997). Localization and neurohemal release of FMRFamide-related peptides in the stick insect *Carausius morosus*.. *Peptides
*.

[bibr25] Moffett SB, Moffett DF (2005). Comparison of immunoreactivity to serotonin, FMRFamide, and SCPb in the gut and visceral nervous system of larvae, pupae, and adults of the yellow fever mosquito *Aedes aegypti*.. *Journal of Insect Science*.

[bibr26] Nambu JR, Murphy-Erdosh C, Andrews PC, Feistner GJ, Scheller RH (1988). Isolation and characterization of a *Drosophila* neuropeptide gene.. *Neuron*.

[bibr27] Nässel DR (2002). Neuropeptides in the nervous system of *Drosophila* and other insects: multiple roles as neuromodulators and neurohormones.. *Progress in Neurobiology*.

[bibr28] Nässel DR, Bayraktaroglu E, Dircksen H (1994). Neuropeptides in neurosecretory and efferent neural systems of insect thoracic and abdominal ganglia.. *Zoological Journal of the Linnean Society*.

[bibr29] Nässel DR (1988). Serotonin and serotonin-immunoreactive neurons in the nervous system of insects.. *Progress in Neurobiology*.

[bibr30] Nichols R (1992a). Isolation and structural characterization of Drosophila TDVDHVFLRFamide and FMRFamide-containing neural peptides.. *Journal of Molecular Neuroscience*.

[bibr31] Nichols R (1992b). Isolation and expression of the *Drosophila* drosulfakinin neural peptide gene product, DSK-1.. *Molecular and Cellular Neuroscience*.

[bibr32] Nichols R (2003). Signaling pathways and physiological functions of *Drosophila melanogaster* FMRFamide-related peptides.. *Annual Review of Entomology*.

[bibr33] Nichols R, Lim IA (1996). Spatial and temporal immunocytochemical analysis of Drosulfakinin (Dsk) gene products in the *Drosophila melanogaster* central nervous system.. *Cell and Tissue Research*.

[bibr34] Nichols R, McCormick J, Lim I (1997). Multiple antigenic peptides designed to structurally related *Drosophila* peptides.. *Peptides*.

[bibr35] Orchard I, Lange AB, Bendena WG (2001). FMRFamide-related peptides: a multifunctional family of structurally related neuropeptides in insects.. *Advances in Insect Physiology*.

[bibr36] Price DA, Greenberg MJ (1977). Structure of a molluscan cardioexcitatory neuropeptide.. *Science*.

[bibr37] Raabe M (1982). *Insect Neurohormones*..

[bibr38] Richer S, Stoffolano JG, Yin C-M, Nichols R (2000). Innervation of Dromyosuppressin (DMS) immunoreactive processes and effect of DMS and benzethonium chloride on the *Phormia regina* crop.. *Journal of Comparative Neurology*.

[bibr39] Schneider LE, Taghert PH (1998). Isolation and characterization of a *Drosophila* gene that encodes multiple neuropeptides related to Phe-Met-Arg-Phe-NH2 (FMRFamide).. *Proceedings of the National Academy of Sciences USA*.

[bibr40] Sivasubramanian P (1991). FMRFamide-like immunoreactivity in the ventral ganglion of the fly *Sarcophaga bullata*: metamorphic changes.. *Comparative Biochemistry and Physiology*.

[bibr41] Sivasubramanian P (1992). Localization of FMRFamide-like immunoreactivity in the larval midgut of the fly, *Sarcophaga bullata*.. *Comparative Biochemistry and Physiology
*.

[bibr42] Stay B, Zhang JR, Kwok RD, Tobe SS (2003). Localization and physiological effects of RFamides in the corpora allata of the cockroach *Diploptera punctata* in relationship to allatostatins.. *Peptides*.

[bibr43] Sternberger LA (1979). *Immunocytochemistry*..

[bibr44] Stoffolano JG (1974). Influence of diapause and diet on the development of the gonads and accessory reproductive glands of the black blowfly, *Phormia regina* (Meigen).. *Canadian Journal of Zoology*.

[bibr45] Stoffolano JG, Dai J-D, Yin C-M (1989). Ultrastructure of the posterior midgut and posterior midgut endocrine cells in *Phormia regina* (Meigen)(Diptera: Calliphoridae).. *Estratto da Redia*.

[bibr46] Tang Y, Ward RD (1998). Sugar feeding and fluid destination control in the phlebotomine sandfly *Lutzomyia longipalpis* (Diptera: Psychodidae).. *Medical and Veterinary Entomology*.

[bibr47] Tsang PW, Orchard I (1991). Distribution of FMRFamide-related peptides in the blood-feeding bug, *Rhodnius prolixus*.. *Journal of Comparative Neurology
*.

[bibr48] Tu M-P (2000). The roles of midgut hormone and allatotropin in the adult black blow fly, Phormia regina Meigen (Diptera: Calliphoridae). *PhD Dissertation*..

[bibr49] Veenstra JA, Lau GW, Agricola H-J, Petzel DH (1995). Immunohistological localization of regulatory peptides in the midgut of the female mosquito *Aedes aegypti*.. *Histochemistry and Cell Biology
*.

[bibr50] White K, Hurteau T, Punsal P (1986). Neuropeptide-FMRFamide-like immunoreactivity in *Drosophila*: development and distribution.. *Journal of Comparative Neurology*.

